# Metabolic Syndrome Among Young Health Professionals in the Multicenter Latin America Metabolic Syndrome Study

**DOI:** 10.1089/met.2019.0086

**Published:** 2020-02-28

**Authors:** Barbara Vizmanos, Alejandra Betancourt-Nuñez, Fabiola Márquez-Sandoval, Laura I. González-Zapata, Julia Monsalve-Álvarez, Josefina Bressan, Fernanda de Carvalho Vidigal, Rafael Figueredo, Laura Beatriz López, Nancy Babio, Jordi Salas-Salvadó

**Affiliations:** ^1^ Cuerpo Académico UDG-454, Alimentación y Nutrición en el proceso Salud Enfermedad, Departamento de Reproducción Humana, Crecimiento y Desarrollo Infantil, Centro Universitario de Ciencias de la Salud, Universidad de Guadalajara, Guadalajara, México.; ^2^ Cuerpo Académico UDG-454, Alimentación y Nutrición en el proceso Salud Enfermedad. Departamento de Disciplinas Filosófico, Metodológico e Instrumentales, Centro Universitario de Ciencias de la Salud, Universidad de Guadalajara, Guadalajara, México.; ^3^ Grupo de Investigación en Determinantes Sociales del Estado de la Salud y la Nutrición, Escuela de Nutrición y Dietética, Universidad de Antioquia, Medellín, Colombia.; ^4^ Escuela de Nutrición y Dietética, Universidad de Antioquia, Medellín, Colombia.; ^5^ Departamento de Nutrición y Salud, Universidad Federal de Viçosa, Viçosa, Brazil.; ^6^ Facultad de Nutrición, Universidad Federal de Alfenas, Alfenas, Brazil.; ^7^ Facultad de Ciencias Médicas, Universidad Nacional de Asunción, Asunción, Paraguay.; ^8^ Escuela de Nutrición, Facultad de Medicina, Universidad de Buenos Aires, Buenos Aires, Argentina.; ^9^ Unidad de Nutrición Humana, Facultad de Medicina y Ciencias de la Salut, IISPV, Universitat Rovira i Virgili, Reus, Spain.; ^10^ CIBER de Fisiopatología de la Obesidad y la Nutrición (CIBEROBN), Instituto de Salud Carlos III, Gobierno de España, Madrid. Spain.

**Keywords:** metabolic syndrome, health professionals, Latin America, dyslipidemia, abdominal obesity

## Abstract

***Background:*** Metabolic syndrome (MS) and its components increase the risk of a number of chronic diseases. Evidence regarding its prevalence among health professionals, particularly in Latin America, is limited. The purpose of this study was to assess the overall prevalence of MS and its components among health professionals and students from five Latin American countries.

***Methods:*** A cross-sectional multicenter study entitled LATIN America METabolic Syndrome (LATINMETS) was conducted on five groups of apparently healthy volunteer subjects. Sociodemographic factors, lifestyle variables (smoking and physical activity), anthropometric measurements (weight, height, and waist circumference), standard biochemical analyses [triglycerides, glucose, and high-density lipoprotein cholesterol (HDL-C)], and blood pressure measurements were assessed. MS was diagnosed based on internationally harmonized criteria. Associations between MS components and sociodemographic, lifestyle, and anthropometric variables were analyzed using multivariate logistic regression.

***Results:*** A total of 1,032 volunteers (*n* = 316-Mexico, *n* = 285-Colombia, *n* = 223-Brazil, *n* = 132-Paraguay, and *n* = 76-Argentina) were recruited. The majority of them were women (71.9%), students (55.4%), and younger than 28 years (67.2%). The overall prevalence of age-standardized MS was 15.5% (23.1% men and 12.2% women). The majority (59.3%) presented at least one MS component, mainly abdominal obesity (29.7%) and low HDL-C levels (27.5%). After adjusting for age and sex, MS and its components were positively associated with being overweight or obese.

***Conclusions:*** MS prevalence in this study was similar to that generally found among young populations in Latin-American countries. More than half of the sample had at least one MS component, suggesting that preventive measures and treatments aimed at achieving low-risk health status are essential in this population.

## Introduction

Several Latin American countries are undergoing a demographic, epidemiological and nutritional transition that has affected the region's disease profile.^1,2^

Noncommunicable diseases—particularly cardiovascular diseases (CVDs), cancer, and type 2 diabetes (DM2)—are now among the leading causes of death (accounting for 70%–80% of all deaths) in most Latin-American countries, including Colombia,^3^ Brazil,^4^ Mexico,^5^ Paraguay,^6^ and Argentina.^7^

Metabolic syndrome (MS) increases the risk of developing CVD and DM2.^8,9^ It is considered to exist when an individual presents three or more of the following risk factors: abdominal obesity (AO), high fasting plasma glucose (High-FPG), hypertriglyceridemia, low levels of high-density lipoprotein cholesterol (HDL-C; low HDL-C), and high blood pressure (High-BP).^10^ The etiology of MS is multifactorial and attributable to an interaction between genetic, metabolic, and environmental factors.^11^ MS prevalence varies in accordance with the age, sex, and ethnic profiles of the populations studied, as well as the criteria used to diagnose it.^11–13^ The populations of several Latin American countries (Chile, Mexico, Venezuela, Ecuador, Puerto Rico, Colombia, Brazil, Peru, Argentina, and Uruguay) have exhibited a high prevalence of MS (>20%),^11,12,14–23^ as assessed by a range of diagnostic criteria. This high prevalence coupled with the condition's health consequences makes MS a significant public health problem in these countries.^24^

Data on MS prevalence among health professionals, particularly in Latin America, are limited, and data on this population based on uniform MS diagnostic criteria are even more scarce. Studies in Mexico suggest that the prevalence of MS within this population (mainly in the fields of medicine and nursing) may be comparable to its prevalence within the general population (>20%).^25–29^ Among health professionals in Brazil^30^ and medical students in Mexico,^31^ Venezuela,^32^ and Ecuador,^33^ prevalence rates under 15% have been reported based on a variety of diagnostic criteria.

Health professionals are an important segment of the population because of the role they play in promoting health. A congruence between what they know and what they do could thus be expected, as could an expectation for this population to maintain a low-risk health status. Evaluating cardiometabolic risk factors among health professionals is important because unhealthy behaviors within this population could negatively influence the health of the general population. Moreover, healthy lifestyles among health professionals have been associated with their patients having positive attitudes toward preventive recommendations^34^ as well as a higher likelihood of engaging in preventive health practices.^35^

Assessments of cardiometabolic risk factors among health professionals could lead to recommendations for the treatment and/or prevention of MS complications that would benefit both this group and the general population.

The purpose of this multicenter study was to evaluate the overall prevalence of MS and its components in a sample of young health professionals from five Latin American countries (Mexico, Colombia, Brazil, Paraguay, and Argentina).

## Materials and Methods

### Study population

A cross-sectional multicenter study entitled LATIN America METabolic Syndrome (LATINMETS) was conducted. The LATINMETS project was coordinated by Universitat Rovira i Virgili in Reus, Spain, and included research groups in Mexico, Colombia, Brazil, Paraguay, and Argentina.

The LATINMETS study population was made up of a nonrandom or convenience sample of individuals between 20 and 59 years of age in apparent good health, who lived in five different cities in five countries. They were either health professionals who worked at a health facility and/or a higher education institution or university students in health-related fields (medicine, nursing, nutrition, dentistry, psychology, pharmaceutical biochemistry, and physical education), who were in their final semesters of coursework. Volunteers were recruited from one or two health care facilities and/or health education institutions in each of the following cities: Buenos Aires (Argentina), Guadalajara (Mexico), Viçosa (Brazil), Medellín (Colombia), and Asunción (Paraguay) in the period from October 2010 to July 2013. Excluded were the following: (1) pregnant or breastfeeding women; (2) people taking steroids; (3) people suffering from illnesses requiring hospitalization at the time of the study; (4) cancer patients or individuals who had had cancer within 3 years before the study; and (5) subjects who did not complete the entire assessment [BP, blood collection, and waist circumference (WC)].

Seven subjects (Mexico *n* = 3, Brazil *n* = 3, and Argentina *n* = 1) were excluded from the analysis due to a lack of data in relationship to MS components. These participants were women with an average age of 31 years (standard deviation 10.4), who presented normal body mass index (BMI). With respect to the assessed components, only one of these participants had AO, and another had low HDL-C (both from Brazil).

The study was designed in accordance with Declaration of Helsinki guidelines and approved by the respective ethics committees of the universities in each country (Colombia: Medical Research Institute Bioethics Committee, Faculty of Medicine, University of Antioquia, certificate 008-29, in addition to approval by the Research Development Committee, certificate 580-23; Mexico: University Center for Health Sciences Committee of Ethics and Research, University of Guadalajara, registry number CI-13909; Argentina: Provisional Committee on Human Ethics, Faculty of Medicine, University of Buenos Aires, *Resolución Consejo Directivo* 2862; Brazil: Ethics Committee on Research with Human Beings, Federal University of Viçosa, registry number 005/2011; and Paraguay: Research Ethics Committee, Faculty of Medical Sciences, National University of Asuncion). All subjects signed an informed consent, and the confidentiality of their personal data was guaranteed.

### Sociodemographic and lifestyle assessment

By means of interviews, data were collected in the following categories: age, sex, country, occupational status (student/professional), health sector, personal medical history, and medication use. Smoking habits were recorded, and data on physical activity (PA) levels were collected by means of a Spanish-language version of the Minnesota Leisure-Time Physical Activity Questionnaire.^36^ Based on reported frequencies and minutes spent per day on PA together with the metabolic equivalents for each activity, daily PA energy expenditures were estimated and categorized in tertiles to facilitate their interpretation. The number of minutes of PA per week was then calculated and classified according to World Health Organization recommendations (cutoff point 150 min/week).

### Anthropometric assessment

Measurements of body weight (scale, 0.1 kg) and height (stadiometer, 0.1 cm) were taken. BMI was calculated (in kg/m^2^), and each subject was classified according to World Health Organization criteria.^37^ WC was measured at the midpoint between the lowermost rib and the upper portion of the iliac crest (fiberglass measuring tape, 0.1 cm). All measurements were performed by the study's research team according to ISAK (International Society for the Advancement of Kinanthropometry) standards.

### BP assessment

Systolic blood pressure (SBP) and diastolic blood pressure (DBP) were measured on both the left and right arms (semiautomatic oscillometer) according to the recommendations of the European Society of Hypertension and the European Society of Cardiology.^38^ Data from the arm that produced the higher SBP and DBP readings were used.^38^

### Biochemical assessments

Blood samples were collected from all participants after a 12-hr overnight fast, and they were distributed in serum and plasma extractions bottles (two of each) labeled with the participant's code. Subsequently, blood samples were centrifuged (2,500 rpm, 4°C, and 10 min) and immediately stored at −80°C. Analyses were carried out in local laboratories. FPG was determined by the glucose oxidase method, and HDL-C and triglyceride concentrations were assessed using the enzymatic colorimetric method.

### Diagnostic criteria for MS

MS was defined based on a consensus statement on diagnostic criteria issued by number of prominent institutions.^10^ Individuals were diagnosed with MS if they had three or more of the following abnormal conditions: AO (WC ≥80 cm in women and ≥90 cm in men); high-BP (SBP ≥130 mmHg and/or DBP ≥85 mmHg); high-FPG (plasma glucose ≥100 mg/dL); low HDL-C (HDL-C <40 mg/dL in men and <50 mg/dL in women); and hypertriglyceridemia (triglycerides ≥150 mg/dL). Taking pharmacological drugs to treat any of these disorders (antihypertensive drug, hypoglycemic drug, nicotinic acid, or fibrates) was also used as an indicator of the corresponding component, with the exception of AO component.^10^

### Statistical analysis

Qualitative variables were reported as numbers, percentages, and 95% confidence intervals (CIs) for a proportion (using a normal distribution or an exact method based on the binomial distribution, as applicable). Comparisons between proportions were carried out using the chi-square or Fisher's exact test. The frequency of MS was standardized by age. The associations between MS and its components and sociodemographic factors and lifestyle variables were determined by logistic regression analysis. *P* < 0.05 was considered statistically significant. Most calculations were performed using SPSS version 25 statistical software for Windows, while the Stata program (version 15) was used for the calculation, which produced the 95% CI for a proportion using an exact method based on the binomial distribution.

## Results

### General characteristics of the sample

A total of 1,032 volunteers were analyzed. The sample consisted of women (71.9%), students (55.4%), and subjects younger than 28 years (67.2%). Overweight (BMI ≥25) was observed in 30.7% of subjects, and 7.5% were smokers. The presence of pathologies such as DM2 (1.0%), dyslipidemia (7.9%), and hypertension (3.0%) was rare, as was taking medication to treat such disorders (<2%). Most participants performed PA more than 150 min/week (94.5%). Significant differences between countries (*P* < 0.05) were observed for all of the previously described variables. A description of general variables by country is shown in Table 1.

**Table 1.** General Characteristics of the Sample by Country

	Total, *n* = 1,032	Mexico, *n* = 316	Colombia, *n* = 285	Brazil, *n* = 223	Paraguay, *n* = 132	Argentina, *n* = 76	P^a^
	n (%)	n (%)	n (%)	n (%)	n (%)	n (%)	
Sex							
Male	290 (28.1)	92 (29.1)	83 (29.1)	58 (26.0)	52 (39.4)	5 (6.6)	0.001^b^
Female	742 (71.9)	224 (70.9)	202 (70.9)	165 (74.0)	80 (60.6)	71 (93.4)	
Age (years)							
≤23	401 (38.9)	179 (56.6)	91 (31.9)	46 (20.6)	37 (28.0)	48 (63.2)	0.001^b^
24–28	292 (28.3)	75 (23.7)	63 (22.1)	86 (38.6)	50 (37.9)	18 (23.7)	
≥29	339 (32.8)	62 (19.6)	131 (46.0)	91 (40.8)	45 (34.1)	10 (13.2)	
Occupational status							
Student	572 (55.4)	188 (59.5)	163 (57.2)	65 (29.1)	80 (60.6)	76 (100.0)	0.001^b^
Professional	460 (44.6)	128 (40.5)	122 (42.8)	158 (70.9)	52 (39.4)	0 (0.0)	
Health sector							
Nutrition	351 (34.1)	78 (24.8)	69 (24.2)	115 (51.6)	15 (11.4)	74 (100.0)	0.001^b^
Medicine	237 (23.0)	41 (13.0)	84 (29.5)	10 (4.5)	102 (77.3)	0 (0)	
Nursing	134 (13.0)	50 (15.9)	56 (19.6)	13 (5.8)	15 (11.4)	0 (0)	
Other areas	307 (29.8)	146 (46.3)	76 (26.7)	85 (38.1)	0 (0)	0 (0)	
BMI							
Underweight	52 (5.0)	22 (7.0)	9 (3.2)	11 (4.9)	5 (3.8)	5 (6.6)	0.001^b^
Normal weight	664 (64.3)	189 (59.8)	188 (66.0)	164 (73.5)	59 (44.7)	64 (84.2)	
Overweight	237 (23.0)	78 (24.7)	73 (25.6)	40 (17.9)	39 (29.5)	7 (9.2)	
Obese	79 (7.7)	27 (8.5)	15 (5.3)	8 (3.6)	29 (22.0)	0 (0.0)	
Smoking status							
Nonsmoker	888 (87.4)	267 (84.8)	253 (88.8)	200 (95.7)	105 (80.2)	63 (82.9)	0.001^b^
Smoker	76 (7.5)	33 (10.5)	11 (3.9)	6 (2.9)	16 (12.2)	10 (13.2)	
Former smoker	52 (5.1)	15 (4.8)	21 (7.4)	3 (1.4)	10 (7.6)	3 (3.9)	
PA (min/week)							
<150	57 (5.5)	6 (1.9)	17 (6.0)	6 (2.7)	23 (17.4)	5 (6.6)	0.001^b^
>150	975 (94.5)	310 (98.1)	268 (94.0)	217 (97.3)	109 (82.6)	71 93.4	
PA energy expenditure (kcal/day)							
≤317	344 (33.3)	94 (29.7)	96 (33.7)	50 (22.4)	68 (51.5)	36 (47.4)	0.001^b^
318–596	344 (33.3)	114 (36.1)	92 (32.3)	82 (36.8)	33 (25.0)	23 (30.3)	
≥597	344 (33.3)	108 (34.2)	97 (34.0)	91 (40.8)	31 (23.5)	17 (22.4)	

Qualitative data are expressed by number (*n*) and percentage (%). The total *n* in the variable “smoking status” is 1016 and the total *n* in the variable “health sector” is 1029.

^a^ Comparisons between proportions were carried out using the chi-square.

^b^ *p* < 0.05.

BMI, body mass index; PA, physical activity.

### Prevalence of MS

The overall prevalence of age-standardized MS was 15.5% (23.1% in men, 12.2% in women). The prevalence of MS increased with age and BMI (*P* < 0.001). Age-standardized MS was most frequent in participants from Paraguay (28.3%) and less frequent in those from Colombia (15.7%), Mexico (13.9%), Brazil (10.4%), and Argentina (3.6%). Likewise, age-standardized MS was more prevalent among health professionals (14.6%), medical professionals and students (24.2%), and those who performed PA <150 min/week (28%) than it was among health students (8.0%), nutrition professionals and students (7.6%), and those whose per-week PA frequency was higher (14.5%). Data for non-age-standardized MS prevalence are shown in Table 2.

**Table 2.** Overall Prevalence of Metabolic Syndrome in Accordance with Sociodemographic Characteristics

	Total	MS prevalence	
	*n*	*n*	% (95% CI)
Total^a^	1032	102	9.9 (8.1–11.7)
Mexico	316	22	7.0 (4.1–9.8)
Colombia	285	37	13.0 (9.1–16.9)
Brazil	223	11	4.9 (2.1–7.8)
Paraguay	132	30	22.7 (15.5–30.0)
Argentina	76	2	2.6 (0.3–9.2)
Sex^a^			
Male	290	52	17.9 (13.5–22.3)
Female	742	50	6.7 (4.9–8.5)
Age (years)^a,b^			
≤28	693	38	5.5 (3.8–7.2)
≥29	339	64	18.9 (14.7–23.1)
Occupational status			
Student	572	38	6.6 (4.6–8.7)
Professional	460	64	13.9 (10.7–17.1)
Health sector^a^			
Nutrition	351	11	3.1 (1.3–5.0)
Medicine	237	43	18.1 (13.2–23.1)
Nursing	134	19	14.2 (8.2–20.2)
Other areas	307	29	9.4 (6.2–12.7)
BMI^a,b^			
Normal weight	716	11	1.5 (0.6–2.4)
Overweight-obese	316	91	28.8 (23.8–33.8)
Smoking status^b^			
Nonsmoker	940	91	9.7 (7.8–11.6)
Smoker	76	10	13.2 (5.4–20.9)
PA (min/week)^c^			
<150	57	11	19.3 (8.7–29.9)
>150	975	91	9.3 (7.5–11.2)
PA energy expenditure (kcal/day)^b^			
≤596	688	70	10.2 (7.9–12.4)
≥597	344	32	9.3 (6.2–12.4)

Presence of three or more of the following risk factors: AO, high-BP, high-FPG, high-TG, or low HDL-C (medication for one of these conditions should be included as a criterion, even when the measurements taken show adequate values). The total *n* in the variable “smoking status” is 1016 and the total *n* in the variable “health sector” is 1029. The prevalence rates reported in this table are not standardized by age. Data are expressed as numbers (*n*), percentages (%), and 95% CI. Comparisons between proportions were calculated using the chi-square or Fisher's exact test, in accordance with testing conditions.

^a^ *P* < 0.001.

^b^ Variables were recategorized into two categories due to the low frequency of MS in some categories.

^c^ *P* < 0.05.

AO, abdominal obesity; BP, blood pressure; CI, confidence interval; high-TG, hypertriglyceridemia; MS, metabolic syndrome.

### Prevalence of MS components

AO (29.7%) and low HDL-C (27.5%) were the most common MS components. AO was significantly more frequent in subjects from Paraguay (56.8%), men (36.9%), smokers (42.1%), medical professionals and students (47.7%), and those who reported a daily PA energy expenditure of <317 kcal (36.3%), and who spent fewer than 150 min/week performing PA (43.9%). Low HDL-C was most prevalent among subjects in Paraguay (65.2%) and medical professionals and students (41.8%); no significant differences were observed according to age and sex. The frequencies of hypertriglyceridemia (15.8%) and high-FPG (10.7%) were highest in Colombia (22.5% and 26.7%, respectively), among men (25.5% and 18.3%, respectively), and among medical professionals and students (21.5% and 16.5%, respectively). In addition, hypertriglyceridemia was more common among former smokers (30.8%). High-BP was most prevalent in subjects from Paraguay (18.9%) and in men (30.0%). AO, hypertriglyceridemia, high-FPG, and high-BP increased with age (*P* < 0.05). All MS components significantly increased with BMI (Table 3).

**Table 3.** Prevalence of Metabolic Syndrome Components in the Presence of Different Variables (*n* = 1,032)

	Total	AO^a^		Low HDL-C^b^		High-TG^c^		High-BP^d^		High-FPG^e^	
	*n*	*n*	% (95% CI)	*n*	% (95% CI)	*n*	% (95% CI)	*n*	% (95% CI)	*n*	% (95% CI)
Total	1032	306	29.7 (26.9–32.4)	284	27.5 (24.8–30.2)	163	15.8 (13.6–18.0)	143	13.9 (11.7–16.0)	110	10.7 (8.8–12.5)
Country											
Mexico	316	86	27.2^f^ (22.3–32.1)	84	26.6^f^ (21.7–31.5)	36	11.4^f^ (7.9–14.9)	52	16.5^g^ (12.3–20.6)	3	0.9^f^ (0.2–2.7)
Argentina	76	1	1.3 (0.03–7.1)	0	0.0	10	13.2 (5.4–20.9)	13	17.1 (8.4–25.8)	3	3.9 (0.8–11.1)
Brazil	223	55	24.7 (19.0–30.4)	42	18.8 (13.7–24.0)	25	11.2 (7.0–15.4)	28	12.6 (8.2–16.9)	16	7.2 (3.8–10.6)
Colombia	285	89	31.2 (25.8–36.6)	72	25.3 (20.2–30.3)	64	22.5 (17.6–27.3)	25	8.8 (5.5–12.1)	76	26.7 (21.5–31.8)
Paraguay	132	75	56.8 (48.3–65.4)	86	65.2 (56.9–73.4)	28	21.2 (14.1–28.3)	25	18.9 (12.2–25.7)	12	9.1 (4.1–14.1)
Sex											
Male	290	107	36.9^h^ (31.3–42.5)	77	26.6 (21.4–31.7)	74	25.5^f^ (20.5–30.6)	87	30.0^f^ (24.7–35.3)	53	18.3^f^ (13.8–22.7)
Female	742	199	26.8 (23.6–30.0)	207	27.9 (24.7–31.1)	89	12.0 (9.6–14.3)	56	7.5 (5.6–9.4)	57	7.7 (5.8–9.6)
Age (years)											
≤23	401	71	17.7^f^ (14.0–21.5)	106	26.4 (22.1–30.8)	41	10.2^f^ (7.2–13.2)	45	11.2^f^ (8.1–14.3)	25	6.2^f^ (3.9–8.6)
24–28	292	75	25.7 (20.6–30.7)	78	26.7 (21.6–31.8)	40	13.7 (9.7–17.7)	25	8.6 (5.3–11.8)	25	8.6 (5.3–11.8)
≥29	339	160	47.2 (41.9–52.5)	100	29.5 (24.6–34.4)	82	24.2 (19.6–28.8)	73	21.5 (17.1–25.9)	60	17.7 (13.6–21.8)
Health sector											
Nutrition	351	48	13.7^f^ (10.1–17.3)	70	19.9^f^ (15.7–24.1)	43	12.3^g^ (8.8–15.7)	28	8.0^f^ (5.1–10.8)	31	8.8^f^ (5.8–11.8)
Medicine	237	113	47.7 (41.3–54.1)	99	41.8 (35.4–48.1)	51	21.5 (16.2–26.8)	36	15.2 (10.6–19.8)	39	16.5 (11.7–21.2)
Nursing	134	51	38.1 (29.7–46.4)	48	35.8 (27.6–44.0)	19	14.2 (8.2–20.2)	21	15.7 (9.4–21.9)	19	14.2 (8.2–20.2)
Other areas	307	94	30.6 (25.4–35.8)	67	21.8 (17.2–26.5)	49	16.0 (11.8–20.1)	57	18.6 (14.2–22.9)	20	6.5 (3.7–9.3)
BMI											
Underweight	52	0	0.0^f^	9	17.3^f^ (6.7–27.9)	1	1.9^f^ (0.05–10.2)	2	3.8^f^ (0.5–13.2)	2	3.8^f^ (0.5–13.2)
Normal weight	664	75	11.3 (8.9–13.7)	152	22.9 (19.7–26.1)	70	10.5 (8.2–12.9)	54	8.1 (6.0–10.2)	47	7.1 (5.1–9.0)
Overweight	237	152	64.1 (58.0–70.3)	80	33.8 (27.7–39.8)	60	25.3 (19.7–30.9)	53	22.4 (17.0–27.7)	49	20.7 (15.5–25.9)
Obese	79	79	100.0	43	54.4 (43.2–65.7)	32	40.5 (29.4–51.6)	34	43.0 (31.9–54.2)	12	15.2 (7.1–23.3)
Smoking status											
Nonsmoker	888	251	28.3^g^ (25.3–31.2)	248	27.9 (25.0–30.9)	133	15.0^h^ (12.6–17.3)	113	12.7 (10.5–14.9)	92	10.4 (8.3–12.4)
Smoker	76	32	42.1 (30.7–53.5)	22	28.9 (18.5–39.4)	13	17.1 (8.4–25.8)	15	19.7 (10.6–28.9)	7	9.2 (2.6–15.9)
Former smoker	52	18	34.6 (21.2–48.0)	13	25.0 (12.8–37.2)	16	30.8 (17.8–43.7)	11	21.2 (9.7–32.6)	10	19.2 (8.1–30.3)
PA (min/week)											
>150	975	281	28.8^g^ (26.0–31.7)	263	27.0 (24.2–29.8)	153	15.7 (13.4–18.0)	133	13.6 (11.5–15.8)	101	10.4 (8.4–12.3)
<150	57	25	43.9 30.6–57.1	21	36.8 (23.9–49.8)	10	17.5 (7.4–27.7)	10	17.5 (7.4–27.7)	9	15.8 (6.0–25.5)
PA energy expenditure (kcal/day)											
≤317	344	125	36.3^h^ (31.2–41.4)	96	27.9 (23.1–32.7)	64	18.6 (14.5–22.7)	52	15.1 (11.3–18.9)	40	11.6 (8.2–15.0)
318–596	344	95	27.6 (22.9–32.4)	89	25.9 (21.2–30.5)	42	12.2 (8.7–15.7)	39	11.3 (8.0–14.7)	38	11.0 (7.7–14.4)
≥597	344	86	25.0 (20.4–29.6)	99	28.8 (24.0–33.6)	57	16.6 (12.6–20.5)	52	15.1 (11.3–18.9)	32	9.3 (6.2–12.4)

Taking pharmacological drugs to treat any of these last four disorders was also used as an indicator of this component. Data are expressed as number (*n*), percentage (%), and 95% CI. The total *n* in the variable “smoking status” is 1016 and the total *n* in the variable “health sector” is 1029. The distribution of cases across qualitative variables was analyzed with a chi-squared or Fisher's exact test, according to testing conditions.

^a^ AO: waist circumference ≥80 cm in women and ≥90 cm in men.

^b^ Low HDL-C: HDL-C <40 mg/dL in men and <50 mg/dL in women.

^c^ High-TG: triglycerides ≥150 mg/dL.

^d^ High-BP: SBP ≥130 mmHg and/or DBP ≥85 mmHg.

^e^ High-FPG: plasma glucose ≥100 mg/dL.

^f^ *P* < 0.001.

^g^ *P* < 0.05.

^h^ *P* < 0.01.

DBP, diastolic blood pressure; FPG, fasting plasma glucose; HDL-C, high-density lipoprotein cholesterol; SBP, systolic blood pressure.

More than half of participants showed one or more MS components (59.3%). The majority presented one (34.7%) or two (14.7%) components, while subjects presenting three (6.6%), four (2.9%), or five (0.4%) components were observed with less frequency.

### Association between MS and its components with sociodemographic characteristics and lifestyle variables

After adjusting for age, being female was negatively associated with MS and all of its components, except low HDL-C. In addition, after adjusting for sex, being older than 29 years was positively associated with MS and every one of its components, except low HDL-C.

After adjusting for age and sex, being a professional was negatively associated with high-BP; being a smoker was positively associated with AO; and being a medical or nursing student or professional was positively associated with MS, AO, and low HDL-C. More generally, being either overweight or obese was positively associated with MS and all of its components (Table 4).

**Table 4.** Association Between Metabolic Syndrome and Its Components with Sociodemographic Characteristics and Lifestyle Variables

	MS^a^	AO	Low HDL-C	High-TG	High-BP	High-FPG
	OR (95% CI)	OR (95% CI)	OR (95% CI)	OR (95% CI)	OR (95% CI)	OR (95% CI)
Sex						
Male	1.0	1.0	1.0	1.0	1.0	1.0
Female	0.3 (0.2–0.5^b^)	0.6 (0.5–0.8^b^)	1.1 (0.8–1.4)	0.4 (0.3–0.6^b^)	0.2 (0.1–0.3^b^)	0.4 (0.2–0.6^b^)
	0.3 (0.2–0.5^b^)^c^	0.7 (0.5–0.9^b^)^c^	1.1 (0.8–1.5)^c^	0.4 (0.3–0.6^b^)^c^	0.2 (0.1–0.3^b^)^c^	0.4 (0.3–0.6^b^)^c^
Age (years)						
≤28	1.0	1.0	1.0	1.0	1.0	1.0
≥29	4.0 (2.6–6.1^b^)	3.3 (2.5–4.4^b^)	1.2 (0.9–1.5)	2.4 (1.7–3.4^b^)	2.4 (1.7–3.5^b^)	2.8 (1.8–4.1^b^)
	3.9 (2.6–6.1^b^)^c^	3.3 (2.5–4.4^b^)^c^	1.2 (0.9–1.5)^c^	2.4 (1.7–3.3^b^)^c^	2.4 (1.7–3.6^b^)^c^	2.7 (1.8–4.1^b^)^c^
Occupational status						
Student	1.0	1.0	1.0	1.0	1.0	1.0
Professional	2.3 (1.5–3.5^b^)	1.9 (1.5–2.5^b^)	0.9 (0.7–1.3)	1.9 (1.3–2.6^b^)	1.5 (1.0–2.1^b^)	1.8 (1.2–2.8^b^)
	0.6 (0.3–1.0)^c^	0.8 (0.5–1.1)^c^	0.8 (0.6–1.2)^c^	1.0 (0.6–1.6)^c^	0.6 (0.3–0.9^b^)^c^	0.8 (0.5–1.4)^c^
Health sector						
Nutrition	1.0	1.0	1.0	1.0	1.0	1.0
Medicine	6.8 (3.4–13.6^b^)	5.7 (3.9–8.6^b^)	2.9 (2.0–4.2^b^)	1.9 (1.3–3.1^b^)	2.1 (1.2–3.5^b^)	2.0 (1.2–3.4^b^)
	4.2 (2.0–8.7^b^)^c^	5.4 (3.5–8.3^b^)^c^	3.1 (2.1–4.5^b^)^c^	1.3 (0.8–2.1)^c^	0.9 (0.5–1.6)^c^	1.1 (0.6–1.9)^c^
Nursing	5.1 (2.4–11.0^b^)	3.9 (2.4–6.2^b^)	2.2 (1.4–3.5^b^)	1.2 (0.7–2.1)	2.1 (1.2–3.9^b^)	1.7 (0.9–3.1)
	3.4 (1.5–7.7^b^)^c^	3.0 (1.8–4.9^b^)^c^	2.2 (1.4–3.4^b^)^c^	0.9 (0.5–1.6)^c^	1.6 (0.9–3.0)^c^	1.2 (0.7–2.3)^c^
Other areas	3.2 (1.6–6.6^b^)	2.8 (1.9–4.1^b^)	1.1 (0.8–1.6)	1.4 (0.9–2.1)	2.6 (1.6–4.3^b^)	0.7 (0.4–1.3)
	1.7 (0.8–3.7)^c^	2.3 (1.5–3.5^b^)^c^	1.2 (0.8–1.8)^c^	0.9 (0.5–1.4)^c^	1.2 (0.7–2.1)^c^	0.4 (0.2–0.7^b^)^c^
BMI						
Normal weight	1.0	1.0	1.0	1.0	1.0	1.0
Overweight-obese	25.9 (13.6–49.3^b^)	23.2 (16.4–32.8^b^)	2.2 (1.6–2.9^b^)	3.7 (2.6–5.3^b^)	4.5 (3.1–6.5^b^)	3.3 (2.2–4.9^b^)
	17.0 (8.8–33.0^b^)^c^	24.3 (16.4–35.8^b^)^c^	2.5 (1.8–3.4^b^)^c^	2.5 (1.8–3.7^b^)^c^	2.5 (1.7–3.8^b^)^c^	2.0 (1.3–3.1^b^)^c^
Smoking status						
Nonsmoker	1.0	1.0	1.0	1.0	1.0	1.0
Smoker	1.4 (0.7–2.8)	1.8 (1.1–2.9^b^)	1.1 (0.6–1.8)	1.1 (0.6–2.0)	1.6 (0.9–2.9)	0.8 (0.4–1.9)
	1.2 (0.6–2.6)^c^	1.7 (1.0–2.8^b^)^c^	1.1 (0.6–1.8)^c^	0.9 (0.5–1.8)^c^	1.3 (0.7–2.5)^c^	0.7 (0.3–1.6)^c^
PA (min/week)						
>150	1.0	1.0	1.0	1.0	1.0	1.0
<150	2.3 (1.2–4.6^b^)	1.9 (1.1–3.3^b^)	1.6 (0.9–2.7)	1.1 (0.6–2.3)	1.3 (0.7–2.7)	1.6 (0.8–3.4)
	1.8 (0.8–3.9)^c^	1.7 (0.9–3.0)^c^	1.5 (0.9–2.7)^c^	0.9 (0.4–1.9)^c^	1.0 (0.5–2.2)^c^	1.3 (0.6–2.8)^c^
PA energy expenditure (kcal/day)						
≤596	1.0	1.0	1.0	1.0	1.0	1.0
≥597	0.9 (0.6–1.4)	0.7 (0.5–0.9^b^)	1.1 (0.8–1.5)	1.1 (0.8–1.5)	1.2 (0.8–1.7)	0.8 (0.5–1.2)
	0.9 (0.6–1.6)^c^	0.7 (0.5–1.0)^c^	1.1 (0.8–1.5)^c^	1.1 (0.7–1.6)^c^	1.1 (0.7–1.6)^c^	0.8 (0.5–1.2)^c^

Data are expressed as OR and 95% CI.

^a^ Presence of three or more of the following risk factors: AO, high-BP, high-FPG, high-TG, or low HDL-C.

^b^ *P* < 0.05 was considered significant.

^c^ Adjusted for age and sex (age was adjusted for sex and sex was adjusted for age). Associations between variables and MS components were calculated using logistic regression.

OR, odds ratio.

## Discussion

The lack of consensus data on the prevalence of MS and its components among health professionals has limited the number of initiatives aimed at preventing metabolic disorders within this population. In this regard, the results of this study will contribute by helping to describe a part of that reality using standardized criteria. The LATINMETS study showed an overall prevalence of age-standardized MS of 15.5%. Almost 60% of its subjects presented one or more MS components, mainly AO and low HDL-C. After adjusting for age and sex, MS and all of its components were positively associated with being overweight or obese.

The overall prevalence of age-standardized MS observed in the LATINMETS study (15.5%) was similar to the prevalence rates found in other studies conducted on young people (18–39 years) in the United States (20.3%)^39^ and in Latin American countries (<22%).^14,17,18,40^ One of those studies was the CARMELA (Cardiovascular Risk Factor Multiple Evaluation in Latin America) multicenter study that evaluated general populations in seven Latin American cities.^14^ The MS prevalence found in this study was close to the prevalence rates reported in a sample of health professionals in Brazil (12.8%)^30^ and among medical students in Mexico (14.5%)^31^ and Ecuador (7.5%).^33^ Nonetheless, the overall MS frequency found in this study was lower than the frequencies observed in young people (20–39 years) within the general population (23.8%)^16,20^ and in health professionals who worked at health institutions in Mexico (>30%),^25–29^ and lower than the 25.8% reported in another multicenter study on South American cities (subjects 35–44 years old).^15^

Similarities between the overall prevalence of MS in this study and the prevalence rates found in the above-mentioned studies may be attributable to the fact that most of the subjects in this study were younger than 28 years (67.2%) and presented low obesity rates (7.7%). As found in other published studies, MS frequency in this analysis was shown to increase with age^14–18,27,30,39^ and BMI.^16,17,19,30,39^

The population group evaluated in this study is exposed to cardiometabolic risk, as the majority (59.3%) presented one or more MS components, the most frequent of which were AO and low HDL-C. The risk of CVD and DM2 increases as the number of components increases,^9^ and the number of components can increase with age. A prospective study on Mexican medical students showed that, based on a 6-year follow-up, their rates of AO and MS had increased significantly.^31^ In addition, AO and low HDL-C are independent risk factors for the development of CVD.^41,42^ These two components were also the most frequently reported MS components in other studies on general populations in Latin American countries such as Mexico,^16,19^ Venezuela,^17^ Argentina, Chile, and Uruguay,^15^ in addition to university students in the United States,^43^ medical students in Ecuador^33^ and Mexico,^31^ and health professionals in Brazil^30^ and Mexico.^26–28^ However, the frequency of AO observed in this study is higher than that reported in university students in the United States (22%)^43^ and in medical students from Mexico (17.8%).^31^ Moreover, the frequency of AO in this study was less than the frequencies observed in medical students from Ecuador (43.2%)^33^ and in young health professionals (74.6% younger than 40 years) in Brazil (55.4%).^30^

However, the frequency of low HDL-C in our study was higher than that observed in university students from the United States (12.6%)^43^ and in young Brazilian health professionals (23.8%)^30^; and it was lower than the frequency reported in Ecuadorian medical students (31.8%)^33^ and in medical students from Mexico (59.1%).^31^

The higher prevalence of AO may be attributable to the fact that Latin American populations are more susceptible to abdominal fat accumulation and to the development of insulin resistance and fatty liver than non-Hispanic white populations.^10,11^ In addition, HDL-C concentrations have been shown to depend on genetic factors.^41^ Another factor that may explain the presence of MS components in young adults (18–30 years of age) may be a lack of healthy lifestyle practices.^44,45^ A review showed that the lifestyle habits of most university students, including those enrolled in health care-related fields, leave much to be desired. More specifically, they have unbalanced and high-calorie diets that often include fast food, they do not perform PA frequently, and they consume high amounts of alcohol, tobacco, and other drugs (mainly marijuana).^44,45^ In other words, a high percentage of students do not apply the knowledge that one might assume their university education would give them.^45^ All of these unhealthy behaviors combined with a genetic predisposition and the presence of overweight are risk factors for the development of MS components.^11,41^ In addition, the food environment to which one is exposed may contribute to either positive or negative health outcomes.^46,47^

The early detection and monitoring of metabolic disorders in young people favor the implementation of preventive measures and treatments aimed at achieving low-risk health status before risk factors accumulate and trigger the development of MS or cardiometabolic diseases. We must be aware that the absence of apparent disease in young adults does not mean absence of risk factors.

The main limitation of this study is that the study sample was not obtained at random or stratified by age and sex, despite the presence of these stipulations in protocols. For logistical reasons and due to the reluctance of some health professionals to be assessed, our sample was made up of volunteer subjects. Nonetheless, we believe that this study represents an important step forward in the assessment of MS and its components in young people working or studying in health-related fields in Latin American countries, a population that is and will continue to play a key role in the health of the general population. One of our study's strengths is the limited number of previous studies on MS and cardiovascular risk factors among health professionals. The need for more cross-sectional studies to assess cardiovascular risk factors in college students has already been identified.^44^ Another strength is the fact that MS was assessed using diagnostic criteria issued by prominent institutions in a consensus statement^10^ that included specific cutoff values for the diagnosis of AO for Central and South American populations. In addition, this study is the first to provide data on metabolic disorders on a young population from these five countries using the same MS criteria.
